# Carbolic Acid

**Published:** 1867-10

**Authors:** William Little

**Affiliations:** Chicago


### CARBOLIC ACID.

By William Little, M.D., Chicago.

Within the last few years the attention of the medical profession has been frequently called to the antiseptic and therapeutic properties of this peculiar substance. Lest some of the readers of the Journal may not be familiar with it, I will state what carbolic acid really is. It is one of the products of the distillation of coal-tar oil. If a portion of crude coal-tar oil be placed in a retort furnished with a thermometer, and the portion which distils over between the temperatures of 300° and 400° F., collected and mixed with a strong solution of caustic potash, and allowed to stand for some time, a whitish, pasty mass is obtained, which, when acted upon by water, is resolved into a light oil and a heavier alkaline liquid. If the latter be drawn off and neutralized by hydrochloric acid, carbolic acid will be disengaged, in an impure state, in the form of oil. By distilling from chloride of calcium, to remove any remaining particles of water, and exposing the distillate to a low temperature, carbolic acid congeals in the form of long, colorless, prismatic crystals, which melt at 35° C., to an oily liquid, boiling at 180° C., and resembling, in many particulars, creosote. It is deliquescent, attracts moisture from the atmosphere, and quickly becomes liquid, and continues so at moderate temperatures. It is known by the names, carbolic acid, phenic acid, phenol, phenic alcohol, and the hydrate of phenyle.

It was discovered by Runge, in 1834, who gave it the name carbolic acid. It possesses remarkable powers as an antiseptic. Most of the disinfecting powders now in use are nothing more than a mixture of carbolic acid and plaster of Paris. It is the opinion of some that this acid is capable of absorbing offensive gases of putrefaction, but a number of experiments show plainly that it has no influence over gases, but it prevents putrefaction and thus obviates the formation of gases.

It has the power of arresting fermentation, produced by organized matter, and also prevents its further development. Its mode of action is not thoroughly understood, but it appears to act by attacking vitality in some mysterious way.

The experiments of Mr. Crooks prove, conclusively, its action on vitality. "Cheese mites were immersed in water, where they lived for several hours, a few drops of a solution of carbolic acid, containing 1 per cent, added to the water, killed them instantly. A few drops of the solution was added to water in which small fish were swimming; it proved fatal in a few minutes. Water containing infusoria was placed under the microscope and a small quantity of a weak solution of carbolic acid was added; it proved fatal, and arrested their movements at once. These animalcules are almost constant attendants of putrefactive fermentation."

Dr. Lemar and other observers state that the vapor of carbolic acid proves fatal to flies, ants and their eggs, lice, bugs, ticks, centipedes, acaria, butterflies, earwigs, woodlice, cockchafers, and other insects of their size. When such animals are killed with carbolic acid, their bodies resist putrefaction for a long time. During the past year, a solution of carbolic acid has been used in many of the medical colleges for preserving anatomical material, for which it has proved a valuable agent.

Dr. Crooks had occasion to employ a variety of disinfectants during the prevalence of cattle plague in England, and found none so efficient as carbolic acid.

The vapor is by no means offensive to the higher classes of animals, and there is comparatively little danger in handling it. Should a portion of the acid, in an undiluted state, come in contact with the integument, it acts as a mild caustic, but if rubbed or washed off, no inconvenience is felt. As a therapeutic agent, it has been most extensively used as an external application.

In sloughing wounds, a solution composed of one part acid to forty parts of water produces the most marvelous effects: it destroys all fetor, facilitates the separation of the slough, and causes the parts beneath to assume a healthy appearance. It seems, also, to have the effect of promoting the growth of healthy granulations, and of hastening the healing process of wounds. It has been used successfully in several forms of skin diseases, *viz.*, lepra, tinea capitis, rupia, and eczema. It has proved a valuable agent in the treatment of hemorrhoidal affections and in fistula. It is a valuable caustic, it only affecting a superficial layer of the tissue to which it is applied, hence its use would be indicated in diphtheria and malignant sore throat. But carbolic acid has also been used internally, with beneficial results. One drop, given in the form of a pill, has checked vomiting when other remedies had failed to produce any effect.

It has been highly recommended in cases of dyspepsia, accompanied with pain in the stomach after eating.

It has also been largely used by many eminent French physicians in the treatment of phthisis. A large number of patients in different stages have been treated, with the most favorable results. The mode of administration is as follows:—"Fifteen drops of the pure acid is dissolved in ℨij. of spirits, and the solution mixed with ℥xxxij. of water; this quantity is administered daily, partly by the stomach and partly by the inhalation of fluids in a pulverized form."

Owing to the great demand for carbolic acid, it is largely adulterated. The article most commonly used for this purpose is coal-tar oil, but it can be easily detected.

Pure carbolic acid is soluble in from 20 to 60 parts of water, or in twice its bulk of a solution of caustic soda, while tar oil is nearly insoluble. Therefore, to test carbolic acid, we have only to put a drachm of it in a bottle, pour on it half a pint of warm water, and shake at intervals for half an hour, when the amount of oily matter will show the impurity. Or dissolve one part of caustic soda in ten parts of carbolic acid, the residue will show the amount of impurity.

